# Morphological adaptations for relatively larger brains in hummingbird skulls

**DOI:** 10.1002/ece3.4513

**Published:** 2018-09-27

**Authors:** Diego Ocampo, Gilbert Barrantes, J. Albert C. Uy

**Affiliations:** ^1^ Department of Biology University of Miami Coral Gables Florida; ^2^ Escuela de Biología Universidad de Costa Rica San José Costa Rica

**Keywords:** braincase, eye socket size, Haller's rule, relative brain size, skull ossification

## Abstract

A common allometric pattern called Haller's Rule states that small species have relatively larger brains and eyes than larger species of the same taxonomic group. This pattern imposes drastic structural changes and energetic costs on small species to produce and maintain a disproportionate amount of nervous tissue. Indeed, several studies have shown the significant metabolic costs of having relatively larger brains; however, little is known about the structural constraints and adaptations required for housing these relatively larger brains and eyes. Because hummingbirds include the smallest birds, they are ideal for exploring how small species evolve morphological adaptations for housing relatively larger brain and eyes. We here present results from a comparative study of hummingbirds and show that the smallest species have the lowest levels of ossification, the most compact braincases, and relatively larger eye sockets, but lower eye/head proportion, than larger species. In contrast to Passerines, skull ossification in hummingbirds correlates with body and brain size but not with age. Correlation of these skull traits with body size might represent adaptations to facilitate housing relatively larger brain and eyes, rather than just heterochronic effects related to change in body size. These structural changes in skull traits allow small animals to accommodate disproportionately larger brains and eyes without further increasing overall head size.

## 1 INTRODUCTION

Relative to body size, brain size is proportionately larger in small species than in large species, a widespread pattern known as Haller's Rule (Rensch, 1948). This allometric pattern is found in a wide range of vertebrate and invertebrate taxa (Eberhard & Wcislo, 2011; Huxley, 1932; Striedter, 2005). For instance, brain and eyes of small birds and mammals scale hypoallometrically with body size (Brooke, Hanley, & Laughlin, 1999; Calder, 1984). Consequently, small animals with relatively large brain and eyes have to deal with the behavioral, physiological, and structural costs of producing and maintaining proportionally large brains (Eberhard & Wcislo, 2011). Indeed, the current evidence suggests a significant increase in energetic costs associated with more nervous tissues (e.g., Kotrschal et al., 2013; Niven & Laughlin, 2008); however, little is known about the structural changes that have evolved to deal with housing relatively larger brains and eyes in small animals (e.g., Niven & Farris, 2012).

To provide space for larger brains, cephalized animals can evolve larger heads or evolve changes in shape or structure of the braincase (Eberhard & Wcislo, 2011). For example, in very small spiders, the brain overflows into the coxae and deforms the sternum, reducing the space for prosomal muscles, and, in miniature insects, portions of their brain extend into the prothorax and even the abdomen (Quesada et al., 2011). Similarly, small salamanders have lost some skull bones, reduced skull ossification, and adjusted the overall skull morphology to accommodate relatively larger brains (Hanken, 1983, 1984). These modifications in small arthropods and vertebrates suggest that relatively large brains impose a series of presumably costly modifications in small animals due to the large space required to accommodate large brains. In small birds, adjustments in the skull to accommodate relatively larger brains without enlarging overall head size are expected to be achieved in at least two ways. First, differential growth of some skull bones (allometric variation) could alter the shape of a skull to accommodate more neural tissue. In this case, having a more spherical braincase can accommodate the brain in a more compact way, with deviations from sphericity requiring more area to house the same volume of neural tissue. Second, evolving thinner or less ossified skulls could provide additional space in the braincase because the second layer of bone grows internally, which may be taking up space available for brain tissue.

Although a correlation between the morphological variation of facial and braincase modules has been identified (Bright, Marugán‐Lobón, Cobbe, & Rayfield, 2016; Marcucio, Young, Hu, & Hallgrimsson, 2011; Young, Linde‐Medina, Fondon, Hallgrimsson, & Marcucio, 2017), most studies on avian skull morphology have explored the ecological and molecular factors underlining covariation with beak morphology (e.g., Abzhanov, Protas, Grant, Grant, & Tabin, 2004; Mallarino et al., 2012; Wu, Jiang, Suksaweang, Widelitz, & Chuong, 2004). In contrast, not much is known about the evolutionary processes determining braincase shape, especially adaptations in the smallest species. For instance, birds with rounded eye sockets have more rounded and flexed brains than those with elongated orbits (Kawabe, Shimokawa, Miki, Matsuda, & Endo, 2013), which should affect the overall skull morphology. The current information on hummingbird skull morphology is restricted to general descriptions of its shape (Zusi, 2013).

**Figure 1:**
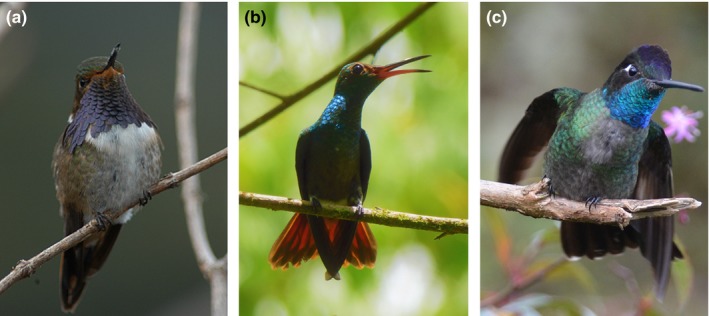
Some species of hummingbirds included in this study are (a) *Selasphorus flammula,* (picture Julio E. Sánchez^†^) (b) *Amazilia tzacatl,* and (c) *Eugenes spectabilis*

Under a phylogenetically controlled comparative framework, we here explore how shape and structure of the skull correlate with body mass and relative brain size in hummingbirds (Figure 1). Hummingbirds include the smallest species of birds and have likely been under strong selection to modify their skull morphology to accommodate larger brains and eyes without enlarging overall head size. We test for the effect of body mass and relative brain size on skull thickness (e.g., degree of ossification), braincase compactness, and relative eye socket size. We predict that small‐bodied hummingbirds, which are known to have relatively larger brains, will have less skull ossification (i.e., single‐layered skull), more compact braincases, and relatively larger eye sockets than large species.

## 2 METHODS

### 2.1 Skull and body measures

We collected the percentage of skull ossification as a proxy for skull thickness from 501 individuals in 96 hummingbird species, from museum specimens in the Louisiana State University Museum of Natural Science (LSUMNS) and Museo de Zoología, Escuela de Biología, Universidad de Costa Rica (MZUCR). Percentage of skull ossification was estimated from ossification pattern, which is the proportion of the braincase having a double layer of bone (i.e., pneumatized) (Harrison & Harrison, 1949; Miller, 1946), as recorded on specimen labels by curators at the moment of skin preparation (Supporting Information Table S1). Likewise, body mass (g) of each individual was taken from specimen labels recorded by curators. We calculated mean values from a sample ranging from 3 to 17 adult males per species, as confirmed by gonads and plumage patterns. For skulls/skeletons without body mass information, we use the mean body mass value for the species to test for the effect of body size on skull compactness (see below). Because brain size of hummingbirds estimated from endocranial volume might be unreliable, we used fresh brain mass and body mass from fresh specimens collected for a subset of 24 species of hummingbirds (Diego Ocampo, César Sánchez, & Gilbert Barrantes data).

**Figure 2:**
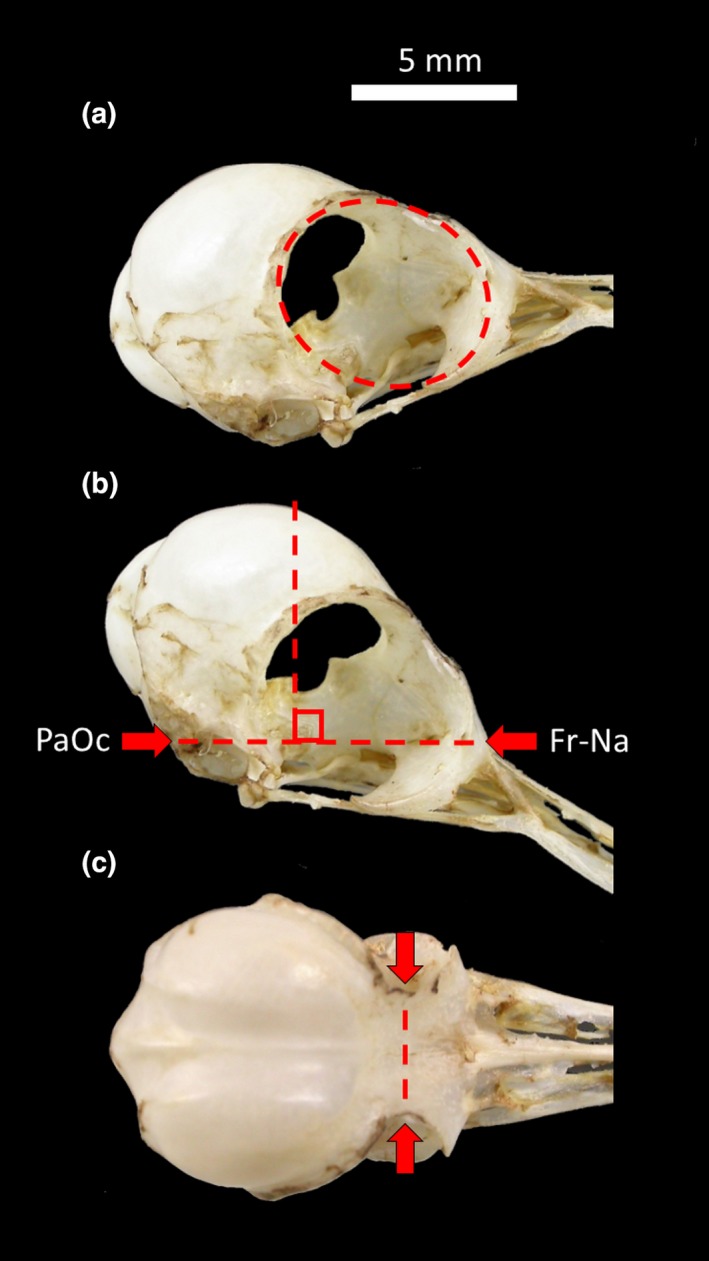
Skull of Rufous‐tailed hummingbird (*Amazilia tzacatl*). (a) The lateral view, with the dashed line representing eye socket area. (b) Lateral view of the skull, with the thick red arrows pointing to the paraoccipital process (PaOc) and the suture between frontal and nasal bones (Fr‐Na). The dashed line represents the angle of the placement of the camera to capture the dorsal view. (c) Dorsal view of the skull, with the red arrows pointing at the most indented region of the frontal bone. The dashed line delimits the anterior border of the braincase

We measured braincase compactness (i.e., circularity) and the area of the contour of the orbit eye socket (eye socket area hereafter) from scaled pictures of skulls for four to six males of 32 species of hummingbirds from the LSUMNS and MZUCR collections (Supporting Information Table S2). We estimated the relative size of the eye socket from pictures of the lateral view of the skull, measuring the ratio between eye socket area and the total area of the skull from the lateral view (without the beak, Figure 2a). We then took pictures of the dorsal view, perpendicularly (90° angle) to the junction between the suture of the frontal and nasal bones, and the paraoccipital process (Figure 2b). From this dorsal view, we estimated the compactness of the braincase based on the ratio between the area and perimeter of the braincase (Peura & Livarinen, 1997). We delimited the frontal border of the braincase by a straight line between the most indented point of the frontal bones at the interorbital region (Figure 2c). We use the compactness (circularity) index as a proxy of the three‐dimensional skull's sphericity. To take standardized pictures, each skull was placed on a small platform, maintaining the camera at the same position relative to either the lateral or the dorsal plane of the skull and at 20 cm from it. All high‐resolution pictures were scaled in coplanarity and analyzed using ImageJ (Abramoff, Magelhaes, & Ram, 2004), and we used the “freehand selection” function to delimit the contour of the braincase and eye socket to measure the areas and perimeters on the pictures.

### 2.2 Statistical analysis

To control for the nonindependence of closely related hummingbird species, we used phylogenetic generalized least square (PGLS) analyses for correlations between variables. We controlled for the phylogenetic relationship, within each subset of species (trees with 96, 32, and 24 hummingbird species, respectively), in each analysis using on 5,000 molecular phylogenies built using the backbone method (Hackett et al., 2008) and data obtained from http://www.birdtree.org (Jetz, Thomas, Joy, Hartmann, & Mooers, 2012). We show the figures generated using the 50% majority rule tree (Supporting Information Figure S1); however, to control for phylogenetic uncertainty, we ran all our analyses using the total population of trees. We report the mean values and standard deviation (±*SD*) of the parameters when pertinent.

To test for the effect of body size or relative brain size on skull ossification, the PGLS models included the log_10_‐transformed body mass or the ratio of brain/body mass as a predictor variable and the log_10_‐transformed percentage of skull ossification as the response variable (log_10_ (percentage of skull ossification + 1), to avoid undefined values). To control for scale effect (i.e., body mass), we ran a PGLS of the residuals of the linear model of the log‐transformed body mass and the log‐transformed brain mass (*x*‐axis), against the residuals of the linear model of the log‐transformed body mass and the log‐transformed skull ossification model (*y*‐axis). A negative correlation would indicate that species with larger brains than expected by body size have skulls with lower ossification than expected.

To test for the effect of body size and skull ossification on braincase compactness, the PGLS model included either the log‐transformed body mass or log‐transformed skull ossification against the mean compactness index (Peura & Livarinen, 1997), as a two‐dimensional proxy of the spherical shape of the braincase. We could not test for the effect of brain size on skull compactness due to the limited number of samples that included both variables (only 11 species) and the extremely high correlation between brain size and body size (*R* ^2^ = 0.94; Diego Ocampo, César Sánchez, & Gilbert Barrantes, unpublished data). We also tested for the effect of log‐transformed body mass on relative eye socket area with a PGLS. We conducted all analyses in R v.3.1.3 (R Core Team, 2014) using the APE (Paradis, Claude, & Strimmer, 2004) and CAPER (Orme, 2013) libraries for the analyses.

## 3 RESULTS

### 3.1 Skull ossification

**Figure 3:**
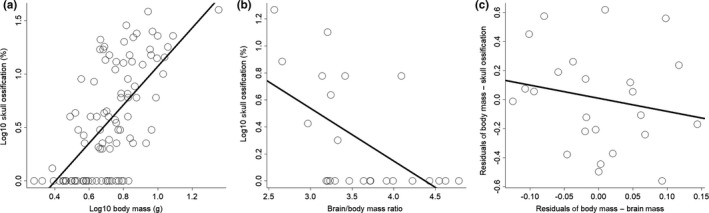
Patterns of skull ossification in hummingbirds. (a) Relationship between body mass and skull ossification for 96 species. (b) Relationship between the brain/body mass ratio and the skull ossification for 24 species. (c) Nonsignificant relationship between the residuals of body mass/brain mass and residuals of body mass/skull ossification (for coefficients and *R* ^2^ values, see main text)

All PGLS models showed a significant effect of body size on skull ossification, with little phylogenetic signal (*F*_1,94_ = 68.13; *p* < 0.001; *R* ^2^ = 0.42; *λ* = 0; Figure 3a). For the 96 species of hummingbirds, the percentage of ossification increased with body size (*β* = 0.78; *p* < 0.001). For the 24 species with brain size data (58 individuals, 1–6 individuals per species), the PGLS models also found an effect of relative brain size on skull ossification (*F*_1,22_ = 9.98; *p* < 0.01; *R* ^2^ = 0.32; *λ* = 0; Figure 3b), in which species with relatively larger brains had lower percentage of ossification (*β* = −0.39; *p* < 0.01). However, when we removed the effect of body size, we did not find a significant effect of brain size on skull ossification (*F*_1,22_ = 0.86; *p* = 0.36; *R* ^2^ = 0.04; *λ* = 0; Figure 3c).

### 3.2 Skull shape

**Figure 4:**
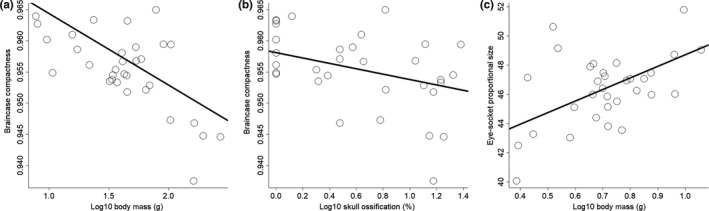
Patterns of skull shape across 32 hummingbird species. Relationship between (a) body mass and braincase compactness. (b) Braincase compactness and skull ossification. (c) Percentage of the skull's lateral area occupied by the eye socket and body size (for coefficients and *R* ^2^ values, see main text)

For a subset of 32 species (five males per species), we found a significant effect of mean body size on mean braincase compactness (*F*_1,30_ = 32.97 ± 1.26; *p* < 0.001; *R* ^2^ = 0.52 ± 0.01; *λ* = 1; Figure 4a): Smaller species had more circular braincases than larger species (*β* = −0.011; *p* < 0.001). In addition, more circular skulls correlated with low levels of ossification (*F*_1,30_ = 10.33 ± 0.42; *β* = −0.004; *p* < 0.005; *R* ^2^ = 0.26 ± 0.01; *λ* = 1; Figure 4b), so that more circular skulls had lower levels of ossification (*β* = −0.0044; *p* < 0.005). Finally, small species had relatively larger eyes (relative to the body) than those of larger species (*F*_1,30_ = 144.44; *β* = 12.55; *p* < 0.001; *R* ^2^ = 0.85; *λ* = 0), but a positive relationship between body size and percentage of the lateral head area occupied by the eye socket (*F*_1,30_ = 10.68; *β* = 3.44; *p* < 0.005; *R* ^2^ = 0.31; *λ* = 0; Figure 4c), with small species having relatively smaller eye sockets (44%) than larger species (50%).

## 4 DISCUSSION

Several studies have explored variation in relative brain size and eye size at various ontogenic and evolutionary scales (Burton, 2008; Linke, Roth, & Rottluff, 1986; Nealen & Ricklefs, 2001), along with their association with ecological and behavioral factors (Dunbar & Shultz, 2007; Maklakov, Immler, Gonzalez‐Voyer, Rӧnn, & Kolm, 2011; Martínez‐Ortega, Santos, & Gil, 2014; Smaers, Dechmann, Goswami, Soligo, & Safi, 2012). However, little is known about how the size of both brain and eyes affects overall skull structure and morphology to accommodate the changes in relative brain and eye size. We found that small hummingbirds had less ossified and more compact or circular braincases and lower eye/head proportion than their large counterparts; these allometric patterns support two nonmutually exclusive hypotheses. First, changes in skull shape and size could be adaptations to accommodate the relatively larger brains of smaller species. That is, changes in skull morphology could result from selection on distinct skeletal modules of the skull, which results in adaptive changes in shape and size to more effectively accommodate larger nervous tissue while mitigating the overall increase in relative head size (“adaptive change hypothesis”). Second, selection acting on body size could change the overall allometric scaling of other structures and organs on body size. For instance, selection favoring small body sizes could also result in small‐bodied species with proportionally larger brains through developmental mechanism (“size‐related constraint hypothesis”).

### 4.1 Adaptive change hypothesis

Direct selection on brain size could affect several skull traits to better accommodate relatively larger brains thus shaping cranial morphology in hummingbirds. For example, the space to encase the relatively large brain and eyes of plethodontid salamanders is limited, imposing structural changes in the surrounding structures (Hanken, 1983). These structural changes include the loss and reduction in skull bones, which results in the brain being partially unprotected but able to house relatively larger brains (Hanken, 1984). Similarly, in hummingbirds, the orbitocranial fonticulus is fused with the optic foramen (Zusi, 2013), resulting in skulls that have an open space between the interorbital septum and the parietal bone.

Increasing compactness or sphericity of the braincase in the smallest species of hummingbird may also allow for housing a relatively larger brain without increasing overall head size. Evolutionary changes in brain size correlate with changes in braincase morphology, since presumably an enlargement of the brain in the early evolution of birds had strong consequences in skull shape (Fabbri et al., 2017). Previous comparative studies across 60 orders of birds have shown that when the brain becomes larger in relation to the cranial base, the braincase becomes more spherical and the foramen magnum is displaced to a more ventral position (Marugán‐Lobón & Buscalioni, 2009). More spherical braincases are associated with species with high flight maneuverability, which likely require more nervous tissue (Iwaniuk & Wylie, 2007). Our results in hummingbirds are consistent with this broadscale study in birds.

In addition, hummingbirds have the highest mass‐specific metabolic rate among vertebrates, which likely represents the upper evolutionary metabolic limit (Suarez, 1992). This high metabolic rate is directly correlated with several physiological and anatomical traits that demand high energy input, such as flight (Lasiewski, 1963) and a relatively large heart (Lasiewski, 1964), brain, and eyes. Therefore, producing and maintaining a disproportionately large amount of nervous tissue, which would include the brain and the retina, which is an outgrowth of the brain itself (Kiltie, 2000), should result in a trade‐off between the relative eye and brain investment (Niven & Laughlin, 2008). Because the smallest hummingbird species may require relatively more space for brain, the space for the eyes may be limited, compared to larger species. Further, the observed changes in the proportion of braincase and eyes could also result from a rearrangement of the three‐dimensional morphological space, rather than changes in volume.

### 4.2 Size‐related constraint hypothesis

Alternatively, selection on traits other than skull morphology, such as body size, might result in secondary nonadaptive changes in skull morphology (McKinney, 1986) because the same developmental pathways link brain morphology and skull characteristics (Koyabu et al., 2014). For example, changes in body size could be achieved through heterochronic changes in the ontogenetic trajectory of the ancestral group (Alberch, Gould, Oster, & Wake, 1979), and skull morphology likely represents adjustments to this new allometric scaling. For instance, the avian skull morphology has likely evolved through a paedomorphic process, because avian skulls retain characteristics of juveniles of ancestral theropods (Bhullar et al., 2012). Similarly, reduced ossification in the smallest of hummingbird species may result from paedomorphosis.

In general, thirteen skeletal traits correlate with body mass in birds (Field, Lynner, Brown, & Darroch, 2013). However, an evolutionary trend of reduction in body size (e.g., miniaturization) has several particular implications for physiological (Eberhard & Wcislo, 2011), behavioral (Cole, 1985; but see Eberhard, 2011), and morphological traits, where the most common morphological outcome is the reduction and fusion of bones (Hanken & Wake, 1993). Thus, if miniaturization has shaped hummingbird skull morphology, hummingbirds may have convergently evolved anatomical traits that are typically found in miniaturized species of other clades, such as relatively larger head and eyes, poorly ossified skeleton, and reductions or loss of bones (Gould, 1977).

Reduced skull ossification in relatively large‐brained species and the general enlargement of the head and eyes found in the smallest species of hummingbirds are comparable with those changes observed in the skull of miniaturized plethodontid salamanders (Hanken, 1983) and Geomyoid rodents (Hafner & Hafner, 1984). The reduction in ossification (i.e., thickness) and the loss of bones are also similar to the drastic changes observed in *Danionella dracula*, a miniaturized cyprinid fish that lacks 44 bones, as a consequence miniaturization (Britz, Conway, & Rüber, 2009). Other examples of changes in skeletal traits correlated with miniaturization, as increased variability and the evolution of morphological novelties, are well documented elsewhere (see Hanken, 1993).

## 5 CONCLUSIONS

The scaling pattern of relative brain size on skull morphological traits and body size is consistent with an evolutionary framework of direct and indirect selection acting on skull morphology. Skull compactness and relative eye socket size likely reflect not just a structural effect of size due to heterochronic changes, but rather adaptations to reduce the costs of housing a relatively large brain and eyes. However, overall head size and changes in skull ossification could have evolved as a consequence of selection on body size. Cichlid fishes show a similar pattern, where the rate of change in brain size with a change in body size is under a strong evolutionary constraint, but species‐specific selective pressures may have shifted the static allometric intercepts (Tsuboi et al., 2016). In essence, these size‐dependent and size‐independent skull traits allow small birds to house relatively larger brain and eyes, without drastically increasing general head size. Overall, our results suggest that paedomorphosis has played an important role in shaping the evolution of hummingbird skulls to more effectively house their relatively large brains. Similar studies in other groups of birds and other vertebrates would provide further insights into the generality of the evolutionary forces shaping adaptations of the skull for housing relatively large brains and eyes.

## AUTHORS’ CONTRIBUTIONS

DO and GB conceived the ideas and designed methodology; DO collected and analyzed the data; DO and JACU led the writing of the manuscript. All authors substantially contributed to the draft and approved the final document for publication.

## DATA ACCESSIBILITY

Skull morphological data is available in the Dryad Digital Repository: https://doi.org/10.5061/dryad.9s364g3.
