# Supplementary material for: Morphological adaptations for relatively larger brains in hummingbird skulls
Source: Ecol Evol. 2018 Sep 27;8(21):10482–8. doi: 10.1002/ece3.4513 (PMC6238128; doi:10.1002/ece3.4513)
Supplement: Supplementary file 1 [file ECE3-8-10482-s001.docx]

**Supporting information Table S1.** Species data for body mass and percentage of skull ossification for 96 species.

| **Species** | ***n*** | **Body mass (g)** | **Skull Ossification (%)** |
| --- | --- | --- | --- |
| *Adelomyia melanogenys* | 5 | 3.94 | 0.0 |
| *Aglaeactis cupripennis* | 3 | 6.30 | 0.0 |
| *Aglaiocercus kingi* | 4 | 4.95 | 12.5 |
| *Amazilia amabilis* | 6 | 4.39 | 0.0 |
| *Amazilia amazilia* | 3 | 4.63 | 20.0 |
| *Amazilia chionogaster* | 5 | 5.10 | 0.0 |
| *Amazilia decora* | 3 | 4.14 | 0.0 |
| *Amazilia edward* | 4 | 4.75 | 1.3 |
| *Amazilia tzacatl* | 10 | 5.04 | 3.5 |
| *Amazilia yucatanensis* | 5 | 4.60 | 2.0 |
| *Anthracothorax nigricollis* | 5 | 7.18 | 21.0 |
| *Archilochus alexandri* | 3 | 2.83 | 0.0 |
| *Archilochus colubris* | 9 | 2.67 | 0.0 |
| *Boissonneaua matthewsii* | 3 | 7.33 | 6.7 |
| *Calypte anna* | 5 | 3.44 | 2.0 |
| *Campylopterus hemileucurus* | 5 | 10.72 | 9.0 |
| *Campylopterus largipennis* | 5 | 9.12 | 14.0 |
| *Chaetocercus mulsant* | 3 | 3.30 | 0.0 |
| *Chalcostigma herrani* | 4 | 5.73 | 2.5 |
| *Chalybura buffonii* | 4 | 6.90 | 1.5 |
| *Chalybura urochrysia* | 3 | 6.76 | 0.0 |
| *Chlorostilbon lucidus* | 5 | 3.08 | 3.0 |
| *Chrysuronia oenone* | 5 | 4.06 | 3.0 |
| *Coeligena coeligena* | 5 | 6.68 | 8.0 |
| *Coeligena iris* | 5 | 6.10 | 5.0 |
| *Coeligena lutetiae* | 4 | 7.18 | 1.3 |
| *Coeligena torquata* | 5 | 7.50 | 5.0 |
| *Coeligena violifer* | 5 | 9.32 | 24.0 |
| *Colibri delphinae* | 5 | 6.68 | 3.0 |
| *Colibri thalassinus* | 7 | 5.20 | 1.4 |
| *Doryfera ludovicae* | 5 | 6.04 | 2.0 |
| *Elvira chionura* | 4 | 3.28 | 0.0 |
| *Ensifera ensifera* | 3 | 9.97 | 21.7 |
| *Eriocnemis alinae* | 3 | 3.83 | 0.0 |
| *Eriocnemis luciani* | 3 | 6.70 | 5.0 |
| *Eriocnemis vestita* | 5 | 4.62 | 1.0 |
| *Eugenes fulgens* | 5 | 9.18 | 2.0 |
| *Eupherusa nigriventris* | 3 | 3.13 | 0.0 |
| *Eutoxeres aquila* | 8 | 11.42 | 16.9 |
| *Eutoxeres condamini* | 5 | 9.88 | 13.0 |
| *Florisuga mellivora* | 9 | 6.66 | 5.6 |
| *Glaucis aeneus* | 3 | 5.23 | 0.0 |
| *Glaucis hirsutus* | 5 | 7.08 | 12.0 |
| *Haplophaedia aureliae* | 5 | 5.16 | 3.0 |
| *Heliangelus amethysticollis* | 4 | 5.73 | 0.0 |
| *Heliangelus exortis* | 4 | 4.70 | 0.0 |
| *Heliangelus micraster* | 5 | 4.88 | 17.0 |
| *Heliangelus regalis* | 4 | 3.75 | 1.3 |
| *Heliodoxa aurescens* | 5 | 6.36 | 19.0 |
| *Heliodoxa branickii* | 5 | 5.72 | 12.0 |
| *Heliodoxa gularis* | 4 | 6.55 | 27.5 |
| *Heliodoxa jacula* | 4 | 8.55 | 1.3 |
| *Heliodoxa leadbeateri* | 5 | 7.54 | 23.0 |
| *Heliodoxa rubinoides* | 4 | 8.15 | 11.3 |
| *Heliodoxa schreibersii* | 5 | 9.00 | 16.0 |
| *Heliomaster furcifer* | 3 | 4.87 | 3.3 |
| *Heliomaster longirostris* | 5 | 6.84 | 14.0 |
| *Heliothryx barroti* | 5 | 5.24 | 0.0 |
| *Hylocharis chrysura* | 4 | 4.25 | 7.5 |
| *Hylocharis cyanus* | 5 | 3.58 | 8.0 |
| *Hylocharis eliciae* | 8 | 3.81 | 0.0 |
| *Klais guimeti* | 4 | 2.80 | 0.0 |
| *Lampornis castaneoventris* | 6 | 5.92 | 0.0 |
| *Lampornis hemileucus* | 4 | 5.75 | 1.3 |
| *Leucippus chlorocercus* | 5 | 4.60 | 16.0 |
| *Metallura eupogon* | 5 | 5.00 | 0.0 |
| *Metallura tyrianthina* | 5 | 3.38 | 0.0 |
| *Microstilbon burmeisteri* | 4 | 1.90 | 0.0 |
| *Ocreatus underwoodii* | 5 | 2.90 | 0.0 |
| *Oreotrochilus melanogaster* | 4 | 8.78 | 37.5 |
| *Panterpe insignis* | 10 | 5.88 | 2.0 |
| *Patagona gigas* | 4 | 22.60 | 38.8 |
| *Phaeochroa cuvierii* | 4 | 9.75 | 5.0 |
| *Phaethornis guy* | 11 | 5.62 | 2.7 |
| *Phaethornis hispidus* | 5 | 4.80 | 16.0 |
| *Phaethornis koepckeae* | 5 | 5.22 | 14.0 |
| *Phaethornis malaris* | 6 | 6.30 | 11.7 |
| *Phaethornis philippii* | 5 | 4.74 | 1.0 |
| *Phaethornis ruber* | 5 | 2.12 | 0.0 |
| *Phaethornis striigularis* | 16 | 2.43 | 0.3 |
| *Phaethornis superciliosus* | 17 | 6.11 | 5.6 |
| *Phaethornis syrmatophorus* | 5 | 5.26 | 1.0 |
| *Polytmus theresiae* | 3 | 3.70 | 1.7 |
| *Pterophanes cyanopterus* | 3 | 10.97 | 13.3 |
| *Ramphomicron microrhynchum* | 3 | 3.70 | 0.0 |
| *Sappho sparganura* | 3 | 5.40 | 0.0 |
| *Schistes geoffroyi* | 5 | 3.66 | 0.0 |
| *Selasphorus flammula* | 12 | 2.47 | 0.0 |
| *Selasphorus platycercus* | 3 | 3.30 | 3.3 |
| *Selasphorus rufus* | 5 | 3.14 | 0.0 |
| *Stellula calliope* | 4 | 2.93 | 0.0 |
| *Thalurania colombica* | 5 | 4.40 | 3.0 |
| *Thalurania furcata* | 14 | 4.51 | 1.1 |
| *Threnetes niger* | 5 | 5.64 | 10.0 |
| *Threnetes ruckeri* | 10 | 6.15 | 0.0 |
| *Topaza pyra* | 3 | 12.23 | 21.7 |

**Supporting information Table S2.** Species data for eye-socket and skull area from the lateral view, the brain case compactness index from the dorsal view and body mass for 32 species. Average body mass per species were calculated on a different set of individuals.

| **Species** | **n “Skulls”** | **Eye-socket**  **(mm^2^)** | **Skull (mm^2^)** | **Braincase area (mm^2^)** | **Braincase perimeter** | **Braincase Compactness** | **n “Mass”** | **Body mass (g)** |
| --- | --- | --- | --- | --- | --- | --- | --- | --- |
| *Adelomya melanogenys* | 4 | 19.69 | 43.61 | 69.59 | 30.69 | 0.96 | 5 | 3.94 |
| *Amazilia amazilia* | 5 | 20.26 | 42.17 | 77.13 | 32.57 | 0.95 | 3 | 4.63 |
| *Amazilia chionogaster* | 5 | 21.89 | 46.41 | 77.94 | 32.77 | 0.95 | 5 | 5.10 |
| *Amazilia tzacatl* | 5 | 28.45 | 59.84 | 66.69 | 30.26 | 0.96 | 10 | 5.04 |
| *Archilochus colubris* | 5 | 17.28 | 36.72 | 51.86 | 26.58 | 0.96 | 9 | 2.67 |
| *Calypte annae* | 5 | 19.41 | 39.60 | 66.90 | 30.22 | 0.96 | 5 | 3.44 |
| *Campylopterus largipennis* | 5 | 30.55 | 62.72 | 111.36 | 39.90 | 0.94 | 5 | 9.12 |
| *Coeligena torquata* | 5 | 28.58 | 60.23 | 100.21 | 37.46 | 0.95 | 5 | 7.50 |
| *Colibri thalassinus* | 6 | 21.03 | 46.05 | 69.06 | 30.85 | 0.95 | 7 | 5.20 |
| *Eugenes fulgens* | 6 | 34.55 | 75.36 | 100.13 | 37.46 | 0.95 | 5 | 9.18 |
| *Eutoxeres aquila* | 5 | 34.74 | 70.92 | 143.70 | 44.95 | 0.94 | 8 | 11.42 |
| *Eutoxeres condamini* | 5 | 34.52 | 66.60 | 139.88 | 44.34 | 0.94 | 5 | 9.88 |
| *Florisuga mellivora* | 6 | 30.54 | 66.50 | 87.55 | 34.32 | 0.96 | 9 | 6.66 |
| *Glaucis aeneus* | 6 | 23.58 | 53.88 | 83.38 | 33.57 | 0.96 | 3 | 5.23 |
| *Glaucis hirsuta* | 5 | 23.62 | 50.22 | 96.55 | 36.30 | 0.96 | 5 | 7.08 |
| *Heliodoxa leabteri* | 4 | 28.14 | 61.13 | 94.42 | 35.90 | 0.96 | 5 | 7.54 |
| *Hylocharis eliciae* | 5 | 21.69 | 50.44 | 58.84 | 28.44 | 0.96 | 8 | 3.81 |
| *Klais guimeti* | 4 | 17.71 | 40.89 | 50.82 | 26.45 | 0.95 | 4 | 2.80 |
| *Leucippus chlorocercus* | 5 | 22.10 | 48.05 | 77.00 | 32.61 | 0.95 | 5 | 4.60 |
| *Metallura eupogon* | 5 | 19.28 | 41.57 | 69.93 | 30.94 | 0.96 | 5 | 5.00 |
| *Panterpe insignis* | 6 | 25.56 | 58.93 | 76.75 | 32.42 | 0.96 | 10 | 5.88 |
| *Phaethornis guy* | 5 | 24.72 | 51.39 | 86.36 | 34.35 | 0.96 | 11 | 5.62 |
| *Phaethornis hispidus* | 5 | 23.07 | 49.20 | 74.90 | 32.16 | 0.95 | 5 | 4.80 |
| *Phaethornis koepki* | 5 | 22.98 | 50.95 | 79.67 | 33.24 | 0.95 | 5 | 5.22 |
| *Phaethornis malaris* | 5 | 25.59 | 54.28 | 95.48 | 36.34 | 0.95 | 6 | 6.30 |
| *Phaethornis philippi* | 5 | 22.50 | 50.77 | 78.03 | 32.78 | 0.96 | 5 | 4.74 |
| *Phaethornis striigularis* | 5 | 17.28 | 43.21 | 47.15 | 25.25 | 0.96 | 16 | 2.43 |
| *Phaethornis superciliosus* | 5 | 26.04 | 55.57 | 94.97 | 36.27 | 0.95 | 17 | 6.11 |
| *Selasphorus flammula* | 5 | 16.49 | 38.69 | 45.17 | 24.73 | 0.96 | 12 | 2.47 |
| *Selasphorus platycercus* | 5 | 16.41 | 32.39 | 58.02 | 28.09 | 0.96 | 3 | 3.30 |
| *Thalurania furcata* | 5 | 21.06 | 43.99 | 74.83 | 32.15 | 0.95 | 14 | 4.51 |
| *Threnetes niger* | 5 | 25.53 | 56.15 | 91.30 | 35.39 | 0.96 | 5 | 5.64 |


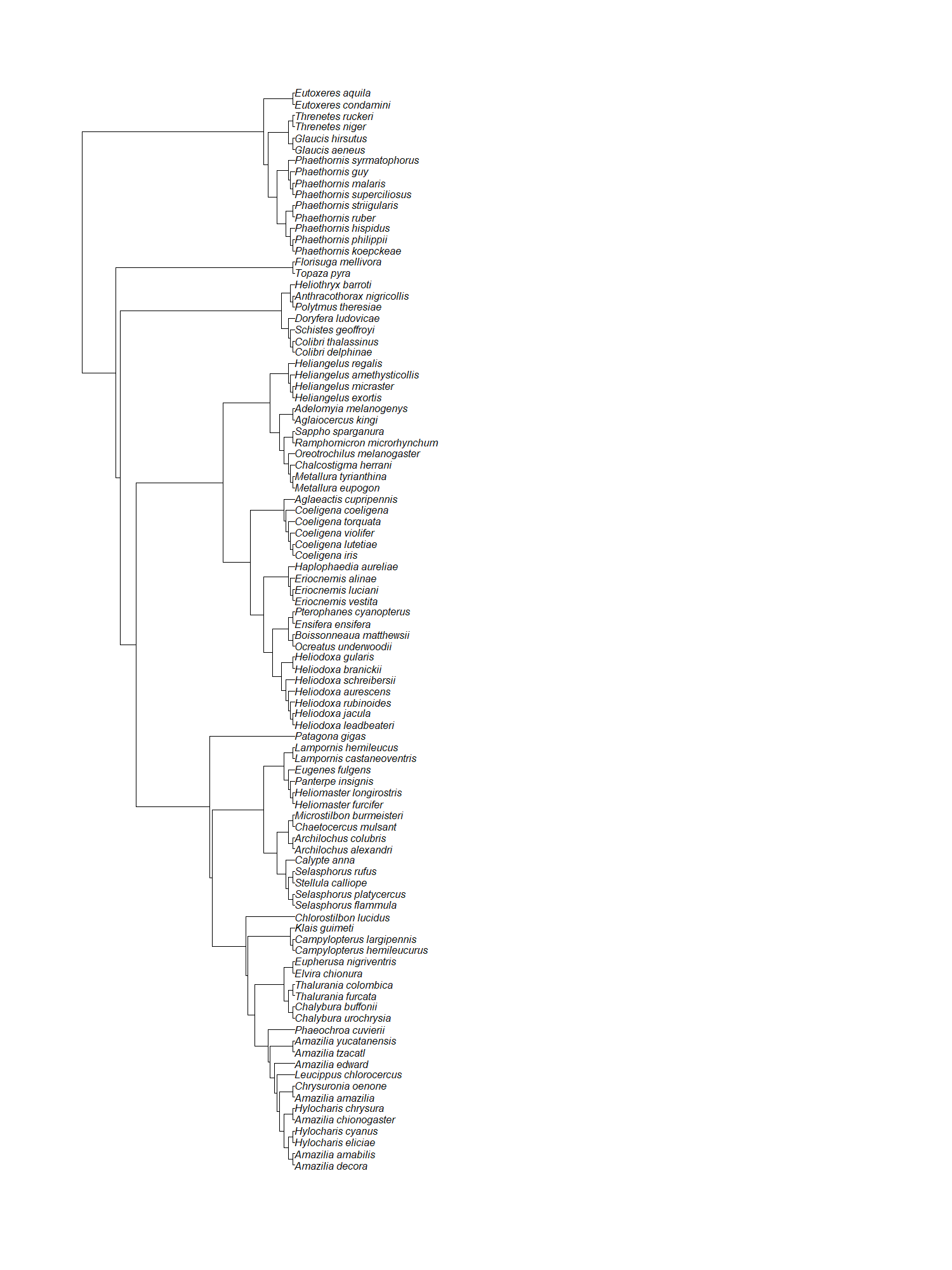


**Supporting information Figure S1.** Consensus tree made using the 50% majority rule, based on 5,000 molecular phylogenies built using a backbone method, trees were obtained from www.birdtree.org (Jetz, Thomas, Joy, Hartmann, & Mooers, 2012).
